# Hsa_circ_0004370 promotes esophageal cancer progression through miR-1294/LASP1 pathway

**DOI:** 10.1042/BSR20182377

**Published:** 2019-05-14

**Authors:** Zhenyang Zhang, Wenwei Lin, Lei Gao, Keqing Chen, Chuangcai Yang, Linwei Zhuang, Shuai Peng, Mingqiang Kang, Jiangbo Lin

**Affiliations:** Department of Thoracic Surgery, Fujian Medical University Union Hospital, Fuzhou City, 350001 Fujian Province, China

**Keywords:** esophageal cancer, hsa_circ_0004370, LASP1, miR-1294

## Abstract

Circular RNAs (circRNAs) formed by back-splicing play multiple roles in the occurrence and development of cancer. Here, we found that hsa_circ_0004370 was up-regulated in both esophageal cancer (EC) tissues and cell lines. Loss function of hsa_circ_0004370 by si-RNA significantly suppressed proliferation and invasion and promoted apoptosis in both EC cell lines. The sponging of miR-1294 by hsa_circ_0004370 was bioinformatically predicted and subsequently verified by luciferase reporter assay and RNA immunoprecipitation assay. Further, hsa_circ_0004370 involved in the up-regulation of LASP1 by sponging miR-1294. Besides, the inhibition of the down-regulated hsa_circ_0004370 on cell malignant behaviors was rescued by miR-1294 inhibitor. Finally, this rescue effect was abrogated by suppressing the expression of LASP1. The results present here suggest that hsa_circ_0004370 functions as an oncogene on cell proliferation, apoptosis, and invasion via miR-1294/LASP1 axis.

## Introduction

Esophageal cancer (EC) is a common malignant tumor in the digestive system. Lifestyle factors, such as smoking, alcohol drinking, and hot beverages, are major risks in the occurrence of EC [1]. Some effective treatments, including surgery, radiation therapy, and chemotherapy, have been clinically applied to improve prognosis [2]. However, most of the patient was diagnosed at the advanced stages and the overall 5-year survival rate of EC was still <20% [3]. Dysregulation of gene expression is critical in the occurrence and development of cancer. Therefore, understanding the potential mechanism involved in the progression of EC is important for developing effective strategies of early diagnosis and therapy.

Numerous factors and molecules are involved in the cancer-related gene dysregulation. Among these molecules, circular RNAs (circRNAs), which were neglected for a long period, are highly conserved RNA molecules formed by back-splicing introns, exons, or intergenic regions [4]. Recent years, dysregulation of circRNAs have been found in many types of cancer [5], and some circRNAs have shown potential applications as biomarkers in the diagnosis of cancer [6,7]. Above all, circRNAs can bind to miRNA to regulate its expression and thus up-regulate or down-regulate the expression of downstream genes [8,9]. In gastric cancer, circ-LARP4 inhibited cell proliferation and invasion through miR-424-5p/LATS1 pathway [4]. Circ-ABCB10 promoted breast cancer proliferation and suppressed cell apoptosis by sponging miR-1271 [10]. Fang et al. [11] proved that cir-ITCH acted as a potential EC suppressor by inhibiting the Wnt/β-catenin pathway through sponging miR-7, miR-17, and miR-214. Even though the functions of some circRNAs have been elucidated, the understanding of circRNAs remains limited and further exploration is imperative.

Hsa_circ_0004370 is located at chr1:170688866-170695542 with a spliced length of 358 in gene symbol PRRX1 (circBase:www.circbase.org/). Previous microarray data and bioinformatic analysis found that hsa_circ_0004370 was significantly up-regulated in a radioresistant esophageal cancer cell and predicted to be involved in the regulation of multiple genes [12]. However, its function in EC progression remains unclear. In the present study, we analyzed the expression of hsa_circ_0004370 in both EC tissues and cell lines. The role of hsa_circ_0004370 in EC cell proliferation, apoptosis and invasion were then explored. Finally, we tried to uncover the potential mechanism of hsa_circ_0004370 involving in EC progression.

## Materials and methods

### Clinical samples

A total of 25 patients without any treatment before surgery were enrolled and the written informed consent was obtained before the operation. The tumor tissues and adjacent normal tissues were collected during surgery and then were snap-frozen in liquid nitrogen quickly and stored at −80°C. All the procedures were proved by the Ethics Committee of Fujian Medical University Union Hospital.

### Cell culture and transfection

Human EC cell lines Eca-109 (primary tumor-derived), TE-1 (primary tumor-derived), and KYSE-150 (primary tumor-derived) were purchased from the Type Culture Collection of the Chinese Academy of Sciences (Shanghai, China). The normal human esophageal epithelial cell line Het-1A was obtained from American Type Culture Collection (Manassas, VA, U.S.A.) and used as a control cell. Cells were cultivated in RPMI-1640 medium (Gibco, Thermo Fisher Scientific, Waltham, MA, U.S.A.) supplemented with 10% fetal bovine serum (FBS) (Thermo Fisher Scientific), streptomycin (100 U/ml) (Thermo Fisher Scientific), and penicillin (100 U/ml) (Thermo Fisher Scientific) at 37°C with an atmosphere of 5% CO_2_. MiR-1294 mimic, miR-1294 inhibitor, specific siRNA target to hsa_circ_0004370 (si- circ_0004370: GCGUCUCCGUACAGAUGACCATT, targeting covalent closed junction) or LASP1 (si-LASP1: GAAUCAAGAAGACCCAGGATT, targeting 458-476), and corresponding controls (si-NC: UUCUCCGAACGUGUCACGUTT) were designed and provided by RiboBio (Guangzhou, China). Cells were cultured till 60–70% confluence and then the transfection was performed using Lipofectamine 2000 (Invitrogen, CA, U.S.A.) as described in manual.

### RNA extraction and quantitative real-time PCR (qRT-PCR)

TRIzol reagent (Invitrogen) was used to extract the total RNA from tissues and cells. cDNA was synthesized using the Prime Script RT Master Mix (Takara, Dalian, China) according to the manufacturer’s instructions. Quantitative real-time PCR (QRT-PCR) was performed using SYBR Premix Ex Taq II (Takara). Mir-X miRNA First Strand Synthesis Kit (Takara) and Mir-X miRNA qRT-PCR SYBR Kit (Takara) were used for the reverse transcription and quantification of miRNA. GADPH and U6 were used as internal control. The expression levels of circ_0004370 and LASP1 were normalized to GADPH and calculated by 2^−ΔΔCt^ method. The expression level of miR-1294 was normalized to U6 and calculated by 2^−ΔΔCt^ method. Primers used here are listed in Table 1.

**Table 1:** Primers used in the present study

Name	Sequence (5′ to 3′)
circRNA F	ACCCACCGATTATCTCTCCTG
circRNA R	TCCTATTCCTTCGCTGCTTTC
GAPDH F	AGAAGGCTGGGGCTCATTTG
GAPDH R	AGGGGCCATCCACAGTCTTC
LASP1 F	CCCAGTCTCCATACAGCGCA
LASP1 R	CTCCACCGTCCCGTACATCC
U6 F	GCTTCGGCAGCACATATACTAAAAT
U6 R	CGCTTCACGAATTTGCGTGTCAT
MiR-1294 F	CGTGTGAGGTTGGCATTGTTGTCT
MiR-1294 R	mRQ 3′ Primer provided by the kit

### Cell proliferation and apoptosis assay

Cells with different treatment were cultivated in 96-well plates (3 × 10^3^ cells per well) at the standard condition for 24, 48, 72, or 96 h. Cell viability was analyzed using Cell Counting Kit-8 (Beyotime, Shanghai, China) at 450 nm as described in manual. For apoptosis assay, cells with different treatment were harvested and washed by PBS buffer. Cells were trypsinized, resuspended in PBS buffer, and labeled by Annexin V-FITC Apoptosis Detection Kit (Beyotime, Shanghai, China) as described in manual and then measured using a flow cytometer.

### Transwell invasion assay

Cell invasion ability was estimated using a Matrigel (BD Biosciences, San Jose, CA, U.S.A.) precoated chamber with 8-μm pore filter (Corning, NY, U.S.A.) in 24-well plates. After transfection, cells were cultured for 24 h and then reseeded in the upper chamber (5 × 10^4^ per well) containing serum-free RPMI-1640 medium. RPMI-1640 medium containing 20% FBS was added to the lower chambers. After 24-h incubation, the cells invaded into the lower chambers were fixed with methanol and stained with Crystal Violet. The number of cells (five fields per chamber) located on the lower chambers was determined by an inverted microscope.

### Western blot analysis

Cells were lysed by RIPA Lysis buffer (Beyotime). Total protein was extracted and quantified by a BCA protein assay kit (Beyotime). Protein samples with equal amounts were separated by SDS-PAGE and transferred onto polyvinylidene fluoride (PVDF) membranes (Sigma-Aldrich, MO, U.S.A.). Subsequently, the PVDF membranes were probed with primary monoclonal antibody against LASP1 (ab156872, 1:5000, diluted with TBS (1% BSA), Abcam, Cambridge, U.K.) or monoclonal antibody against GADPH (ab181602, 1:2000, diluted with TBS (1% BSA), Abcam) overnight at 4°C after blocked by 5% non–fat milk. Finally, the membranes were incubated with the secondary antibody (ab205718, 1:2000; Abcam) and the bands were developed using the EasyBlot ECL kit (Sangon Biotech, Shanghai, China) as described in manual.

### Luciferase reporter assay

The wild-type hsa_circ_0004370 and its mutants were inserted into pmirGLO luciferase vector (Promega, Madison, WI, U.S.A.) to create recombinant plasmids. Similarly, the 3′-UTR regions of LASP1 containing potential binding sites and their mutants were also inserted into pmirGLO luciferase vector. The obtained wild-type and mutant plasmids were cotransfected with miRNA mimic or miRNA NC using Lipofectamine 2000 (Invitrogen) as described in manual. The relative firefly luciferase activity to renilla luciferase activity in the cells was estimated using the Dual-luciferase Reporter Assay System (Promega) according to the manual.

### RNA immunoprecipitation (RIP) assay

Cells were harvested and washed. Subsequently, cell lysis and RNA immunoprecipitation (RIP) assay were performed by Magna RIP RNA-Binding Protein Immunoprecipitation Kit (Merk Millipore, Darmstadt, Germany) as described in manual. The magnetic beads were precoated with antibody against human Ago2 (Millipore) and antibody against IgG (Millipore) was set as an input control. The abundance of target RNAs in bound fractions was subsequently determined by qRT-PCR.

### Statistical analysis

Data analysis was performed by SPSS 19.0 (SPSS Inc., Chicago, U.S.A.). Difference between two groups or multiple groups was analyzed by the Student’s *t*-test or ANOVA. The association of gene expression with clinical characteristics was analyzed using a chi-square test and Fisher’s Exact Test. *P* < 0.05 was considered statistically different. All the experiments were performed in triplicate.

## Results

### Hsa_circ_0004370 was up-regulated in EC tissues and cells

**Figure 1:**
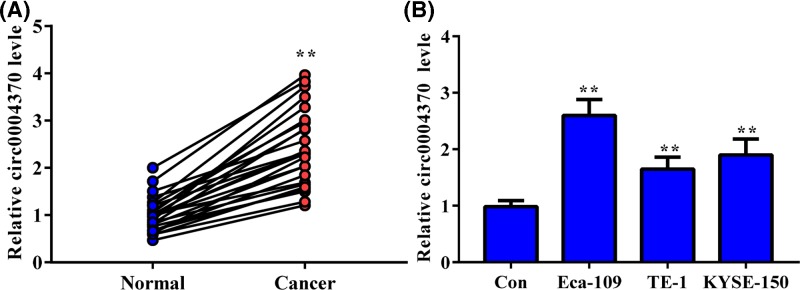
Hsa_circ_0004370 was up-regulated in EC tissues and cells (**A**) Relative expression of hsa_circ_0004370 in EC tissues and the paired adjacent normal tissues were determined by qRT-PCR. (**B**) Relative expression of hsa_circ_0004370 in EC cells were determined by qRT-PCR. Het-1A cell was set as control. Data are represented as mean ± SD; **P<0.05.

We collected the clinical EC tissues and the adjacent normal tissues to analyze the cirRNA level of hsa_circ_0004370. As shown in Figure 1, the expression level of hsa_circ_0004370 significantly higher in the tumor tissues than in the adjacent normal tissues. The median of hsa_circ_0004370 expression level was then set as the cut-off value. High expression of hsa_circ_0004370 was associated with the tumor size in EC (Table 2). Therefore, up-regulation of hsa_circ_0004370 is associated with the malignant progress of EC. The relative expression level of hsa_circ_0004370 in EC cells (Eca-109, TE-1 and KYSE-150) was also up-regulated. The expression level of hsa_circ_0004370 in Eca-109 cell was 2.6-fold of that in Het-1A cell, which was highest level among the tested cells. These results suggest that hsa_circ_0004370 was up-regulated in both EC tissues and cell lines. Eca-109 and KYSE-150 cells with relative higher hsa_circ_0004370 level were then chosen for further study.

**Table 2:** Correlation between circ_0004370 and clinicopathologic features of patients

Clinicopathologic factors	All patients	circ_0004370 expression		*P*
		Low level	High level	
**Age**				
≤60	8	6	5	0.695
>60	17	6	8	
**Gender**				
Male	14	9	6	0.226
Female	11	3	7	
**Stage**				
I+II	13	5	8	0.433
III+IV	12	7	5	
**Size (cm)**				
≤4	10	8	2	0.015
>4	15	4	11	

### Effect of hsa_circ_0004370 on the oncogenic behaviors of EC cells

**Figure 2:**
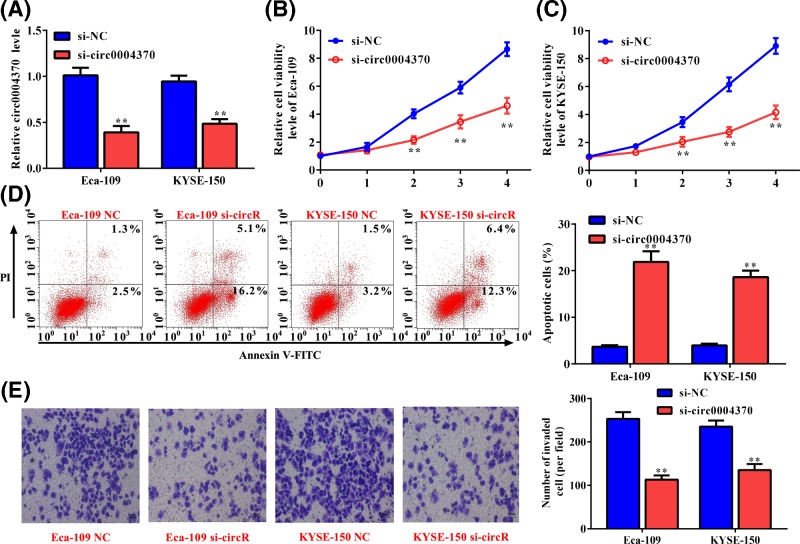
Effect of hsa_circ_0004370 down-regulation on the malignant behaviors of EC cells (**A**) The expression of hsa_circ_0004370 was suppressed by si-RNA. (**B** and **C**) Effect of hsa_circ_0004370 down-regulation on cell viability of Eca-109 and KYSE-150 was determined by CCK-8 assay. (**D**) Effect of hsa_circ_0004370 down-regulation on cell apoptosis was determined by Annexin V/PI analysis. (**E**) Effect of hsa_circ_0004370 down-regulation on cell invasion was determined by Transwell assay. Data are represented as mean ± SD; **P<0.05.

To explore the role of the up-regulated hsa_circ_0004370 in EC, the specific siRNA targeting hsa_circ_0004370 was transformed into Eca-109 and KYSE-150 cells. Transformation of siRNA significantly decreased the cirRNA level by 61% and 51% in Eca-109 and KYSE-150 cells (Figure 2A), respectively, indicating that the expression of hsa_circ_0004370 in both cells were suppressed. Then, we estimated the changes in cell proliferation by CCK8 assay. As shown in Figure 2B,C, cell viabilities of Eca-109 and KYSE-150 were obviously repressed by 46% and 53%, respectively, after the transfection of specific siRNA. Flow cytometry analysis found that the repressed hsa_circ_0004370 level improved the cell apoptosis of Eca-109 and KYSE-150 by more than 4-fold (Figure 2D), indicating that higher cirRNA level promoted cell apoptosis. Besides, Transwell assay showed that hsa_circ_0004370 knockdown obviously inhibited cell invasion of Eca-109 and KYSE-150 by 55% and 47%, respectively (Figure 2E). These results indicate that hsa_circ_0004370 functions as a tumor promoter in EC by promoting cell proliferation and invasion and inhibiting cell apoptosis.

### Hsa_circ_0004370 directly inhibiting miR-1294

**Figure 3:**
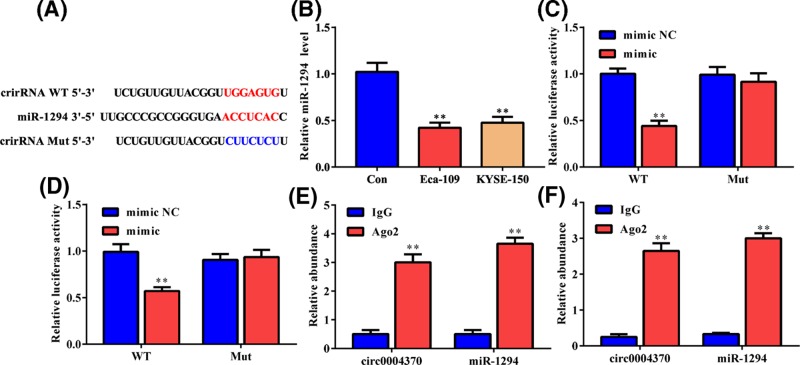
MiR-1294 was a direct target of hsa_circ_0004370 (**A**) Online prediction of the potential binding sites between hsa_circ_0004370 and miR-1294. (**B**) MiR-1294 was down-regulated in both EC cells. (**C** and** D**) The interaction between hsa_circ_0004370 and miR-1294 in Eca-109 and KYSE-150 cells was determined by luciferase reporter assay. (**E** and **F**) The enrichments of hsa_circ_0004370 and miR-1294 in Eca-109 and KYSE-150 cells were performed by RIP assay and then quantified by qRT-PCR. Data are represented as mean ± SD. **P<0.05.

Potential target miRNAs of hsa_circ_0004370 was analyzed by CircInteractome for revealing its mechanism in carcinogenesis. MiRNA-1294 was predicted as a direct target of hsa_circ_0004370 (Figure 3A), and it was down-regulated by more than 50% in both cells (Figure 3B). We applied dual-luciferase reporter assay to confirm the potential binding between hsa_circ_0004370 and miRNA-1294. As shown in Figure 3C,D, miR-1294 mimic significantly decreased the luciferase activities of Eca-109 and KYSE-150 cells containing the wild-type hsa_circ_0004370. However, it had no impact on that of the cells harboring the hsa_circ_0004370 mutant, suggesting the direct binding between hsa_circ_0004370 and miR-1294 in both cells. RIP assay showed that anti-Ago2 antibodies obviously enriched hsa_circ_0004370 and miRNA-1294 than the IgG group in both cells (Figure 3E,F), further confirming the relationship between the two RNA molecules. These results suggest that hsa_circ_0004370 can directly bind to miR-1294 and down-regulate its expression in EC cells.

### Hsa_circ_0004370 promoted LASP1 expression via sponging miR-1294

**Figure 4:**
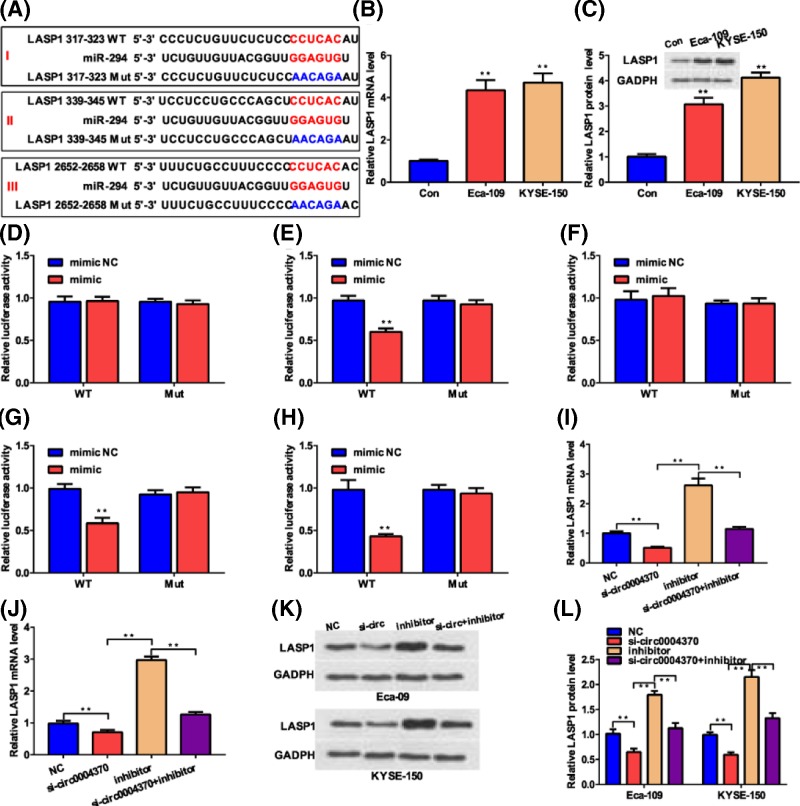
Hsa_circ_0004370 promoted LASP1 expression by sponging miR-1294 (**A**) Online prediction of the potential binding sites between miR-1294 and LASP1. (**B**) Relative mRNA levels of LASP1 in both EC cells were determined by qRT-PCR. (**C**) Relative protein levels of LASP1 in both EC cells were determined by WB assay. (**D**–**F**) The potential interactions between miR-1294 and LASP1 in sites I, II, and III were determined by luciferase reporter assay in HEK293 cells. (**G** and **H**) The potential interactions between miR-1294 and LASP1 in sites II were determined by luciferase reporter assay in Eca-109 and KYSE-150 cells. (**I** and **J**) Relative mRNA levels of LASP1 in Eca-109 and KYSE-150 cells transfected with different molecules were determined by qRT-PCR. (**K**) Relative protein levels of LASP1 in Eca-109 and KYSE-150 cells transfected with different molecules were determined by WB assay. (**L**) Quantitative analysis of the changes in protein levels. Data are represented as mean ± SD; **P<0.05.

Online prediction by Targetscan found that the LIM and SH3 Protein 1 (LASP1) is a potential direct downstream target of miR-1294 (Figure 4A). QRT-PCR analysis and Western blotting found that the mRNA and protein levels of LASP1 was higher in EC cell lines than in the control cell (Figure 4B,C), indicating the up-regulation of LASP1 EC cell lines. We further analyzed the direct regulation relationship between miR-1294 and LASP1 by dual-luciferase reporter assay. As shown in Figure 4A, there were three potential binding sites between the two molecules. The dual-luciferase reporter assay in HEK293 cells showed that miR-1294 can only directly bind to the 339-345 sites of the 3′UTR of LASP1 (Figure 4D–F). Further analysis in Eca-109 (Figure 4G) and KYSE-150 (Figure 4H) cells found that transfection of miR-1294 mimic inhibited the luciferase activity of the cells containing the wild-type sequence, confirming that miR-1294 binds to the 3′UTR of LASP1 at site II. Besides, miR-1294 inhibitor dramatically improved the mRNA level of LASP1 in Eca-109 and KYSE-150 cells by more than 2-fold (Figure 4I,J). These results prove that miR-1294 can directly bind to the 3′-UTR of LASP1 and negatively regulate its expression. To estimate the regulation of LASP1 by hsa_circ_0004370, si-hsa_circ_0004370 and miR-1294 inhibitor were transfected. QRT-PCR found the transfection of si-hsa_circ_0004370 led to lower mRNA level of LASP1 in both cells (Figure 4I,J), indicating that hsa_circ_0004370 positively regulating LASP1. In contrast with the si-hsa_circ_0004370 and miR-1294 inhibitor groups, cotransfection of the two molecules obviously attenuated the changes in the mRNA level of LASP1 in both cancer cells (Figure 4I,J), confirming that hsa_circ_0004370 positively regulating LASP1 through down-regulating miR-1294. Western blotting showed similar changes in the protein level of LASP1 in both cells after transfection of these molecules (Figure 4K,L). These results indicate that hsa_circ_0004370 facilitates the expression of LASP1 via sponging miR-1294 in EC cells.

### Hsa_circ_0004370 facilitated oncogenic behaviors of EC cells via miR-1294/LASP1 pathway

**Figure 5:**
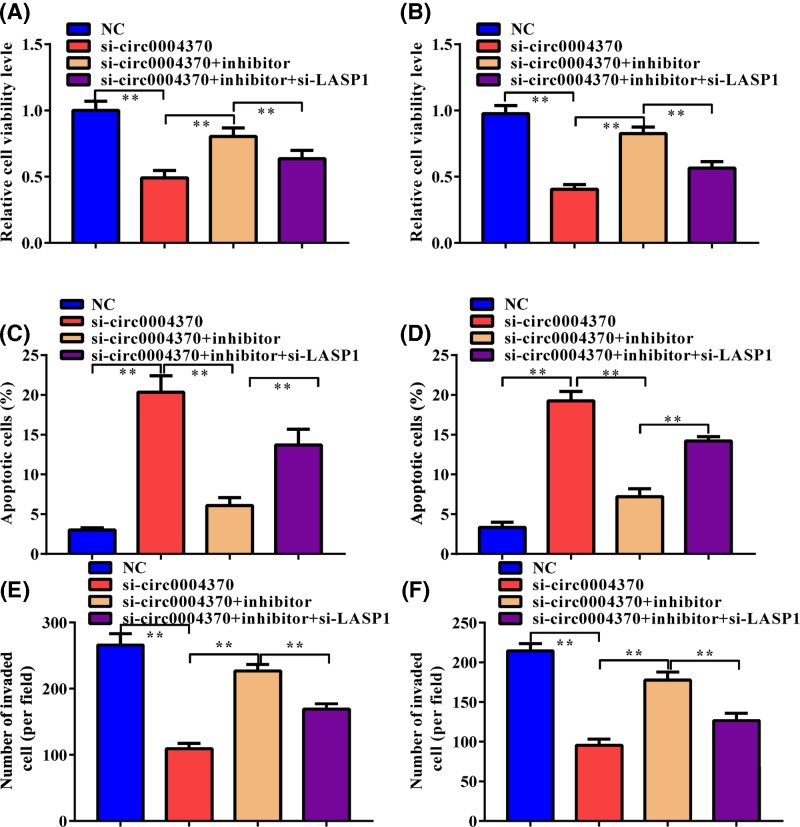
Hsa_circ_0004370 promoted EC cell malignant behaviors via miR-1294/LASP1 axis (**A** and **B**) Effects of si-circ_0004370, miR-1294 inhibitor, or their combination on cell proliferation of Eca-109 and KYSE-150 cells were determined by CCK-8 assay. (**C** and **D**) Effects of si-circ_0004370, miR-1294 inhibitor, or their combination on cell apoptosis of Eca-109 and KYSE-150 cells were determined by Annexin V/PI analysis. (**E** and **F**) Effects of si-circ_0004370, miR-1294 inhibitor, or their combination on cell invasion of Eca-109 and KYSE-150 cells were determined by Transwell assay. Data are represented as mean ± SD; **P<0.05.

We further estimated the role of hsa_circ_0004370/miR-1294/LASP1 axis in the oncogenic behaviors of EC cells. MiR-1294 inhibitor and si-LASP1 were then cotransfected with si-hsa_circ_0004370 in both cells. MiR-1294 inhibitor partly rescued the suppression of si-hsa_circ_0004370 to cell viability (Figure 5A,B), apoptosis (Figure 5C,D), and invasion (Figure 5E,F), suggesting that hsa_circ_0004370 functions as a tumor promoter through miR-1294. Besides, the effect of miR-1294 inhibitor on these oncogenic behaviors of EC cells was further weakened by si-LASP1. These results demonstrate that hsa_circ_0004370 facilitates oncogenic behaviors of EC cells via miR-1294/LASP1 pathway.

## Discussion

Covalently closed loop structure of circRNAs makes it more stable than traditional linear RNAs against RNase and exonuclease to exert its biological function for a long period of time [13]. Development of high-efficient sequencing and bioinformatic analysis technologies has promoted the identification and functional study of circRNAs in recent years. Many circRNAs have been identified and their aberrant expression has been proved to involve in the progression of bladder cancer [14], breast cancer [15,16], lung cancer [17], pancreatic ductal cancer, and cholangiocarcinoma [18] as tumor suppressor or promoter. Previous results implied the tumor promoter role of hsa_circ_0004370 in EC [12]. Here, we found that hsa_circ_0004370 was up-regulated in both clinical EC tissues and cell lines. Further, hsa_circ_0004370 knockdown obviously decreased cell proliferation and migration and promoted cell apoptosis. These results confirm that hsa_circ_0004370 is up-regulated and acts as an oncogenic molecule in EC.

CircRNAs participate in biological processes via multiple mechanisms, including binding RBPs, sponging miRNA, and encoding peptides [19–22]. Thereinto, promoting miRNA degradation through binding to miRNA is the widely studied biofunction of circRNA [22]. MiRNA-1294 was predicted as a potential direct target of hsa_circ_0004370 by online analysis and further luciferase reporter and RIP assays verified the direct binding between hsa_circ_0004370 and miR-1294 in EC cells. Besides, we found that miR-1294 inhibitor attenuated the tumor inhibitory effect of si-circ0004370 in cell proliferation, apoptosis, and invasion. The antitumor function of miR-1294 has been reported in previous studies. In glioma, miR-1294 inhibited cell proliferation and contributed to chemosensitivity via directing TPX2 [23]. Similarly, it was also down-regulated in oral squamous cell carcinoma tissues and cells and acted as a tumor suppressor via inhibiting proliferation and migration through regulating c-Myc [24]. Notably, previous study reported the correlation between the down-regulation of miR-1294 and the poor prognosis of esophageal squamous cell carcinoma [25]. Our results provided another evidence about the role of miR-1294 in EC. Even though the antitumor function of miR-1294 has been illustrated in some reports, the potential mechanism on the dysregulation of the miR-1294 remains unclear. Here, we provided novel information that hsa_circ_0004370 directly down-regulated miR-1294 in EC cells.

Dysregulation of protein expression via different mechanisms plays a key role in the development of cancer. Therefore, the downstream protein regulated by miR-1294 was further explored. LASP1 was predicted as a potential target of hsa_circ_0004370/miR-1294 pathway, which was then verified in both tested EC cells by luciferase reporter assay. LASP1 is an actin-binding protein with an N-terminal LIM domain and the SH3 domain at the C terminus [26]. Although the exact biological function of LASP1 is still ambiguous, the activation of PI3K/AKT [27], MAPK [28], and Smad signaling pathways [29] by LASP1 in cancer progression has been elucidated. LASP1 was also up-regulated and functioned as an oncogenic protein in many malignant tumors [27,28,30,31] by regulating cell proliferation and metastasis [32], cell cycle transition [33], and apoptosis [34]. Our further analysis found that LASP1 involved in hsa_circ_0004370/miR-1294 pathway as a tumor activator through promoting cell proliferation and invasion and inhibiting apoptosis. These result provided novel evidence about the regulation of LASP1 as an oncogenic factor in EC.

In conclusion, the results presented here demonstrate that hsa_circ_0004370 was up-regulated in EC tissues and cells. Further, it functioned as a tumor promoter by promoting cell growth and invasion and repressing cell apoptosis via sponging miR-1294 to repress LASP1.

## Availability of data and materials

The analyzed data sets generated during the study are available from the corresponding author on reasonable request.

## Abbreviations

circRNA: circular RNA
EC: esophageal cancer
RIP: RNA immunoprecipitation
